# Evaluation of Inflammatory Markers in Predicting Coronary Complexity: Insights from the SYNTAX II Score in NSTEMI Patients

**DOI:** 10.3390/jcm13195940

**Published:** 2024-10-06

**Authors:** Murat Bilgin, Emre Akkaya, Recep Dokuyucu

**Affiliations:** 1 Department of Cardiology, Private Aktif International Hospital, Yalova 77720, Turkey; drbilginmurat61@gmail.com; 2 Department of Cardiology, Bossan Hospital, Gaziantep 27580, Turkey; dremreakkaya@hotmail.com; 3 Department of Physiology, Medical Specialization Training Center (TUSMER), Ankara 06230, Turkey

**Keywords:** non-ST-elevation myocardial infarction, neutrophil-to-lymphocyte ratio, monocyte-to-lymphocyte ratio, monocyte-to-HDL-C ratio

## Abstract

**Objectives**: Non-ST-elevation myocardial infarction (NSTEMI) is characterized by the absence of pathological ST segment elevation but an increase in biological markers. The SYNTAX II score (SS-II) is calculated to evaluate the complexity of coronary artery disease and to guide treatment decisions between percutaneous coronary intervention (PCI) and coronary artery bypass grafting (CABG). The aim of this study is to evaluate the relationship of socio-demographic data and biochemical markers with SS-II in NSTEMI patients. **Materials and Methods**: Six hundred patients who were admitted to the private Aktif International Hospital cardiology clinic between January 2020 and January 2024 and were diagnosed with NSTEMI were included in the study. Severity, extent, and clinical evaluation of atherosclerosis were determined using risk factors, laboratory tests, and coronary angiography. Patients were divided into two groups according to their SS-II score: low (≤ 22) and high SS-II (> 32). Socio-demographic data, neutrophil-to-lymphocyte ratio (NLR), monocyte-to-lymphocyte ratio (MLR), and monocyte-to-HDL-C ratio (MHR) were compared between the two groups. **Results**: Group 1 (SS-II ≤ 22) included 380 patients, and group 2 (SS-II > 32) included 220 patients. There was a statistically significant difference in HDL, creatine value, white blood cell, troponin I, hs-TnT, and monocyte values in group 2 compared with group 1 (*p* = 0.001, *p* = 0.018, *p* = 0.031, and *p* = 0.001, respectively). NLR, MLR, MHR, and SS values were statistically significantly higher in group 2 compared with group 1 (*p* = 0.015, *p* = 0.002, *p* = 0.001, and *p* = 0.001, respectively). The risk factors were found to be significantly associated with high-risk NSTEMI (SS-II > 32) in a logistic regression analysis and included peripheral artery disease (PAD) (OR: 3.028, *p* = 0.040), troponin I (OR: 3.575, *p* = 0.015), hs-TnT (OR: 4.221, *p* = 0.010), NLR (OR: 1.528, *p* = 0.024). MLR (OR: 5.248, *p* = 0.012), and MHR (OR: 7.122, *p* = 0.010). ROC analysis revealed that NLR (AUC: 0.691, *p* = 0.016), MLR (AUC: 0.731, *p* = 0.004), and MHR (AUC: 0.824, *p* = 0.003) had higher predictive power than other parameters in patients with high-risk NSTEMI (SS-II > 32). **Conclusions**: We found that NLR, MLR, and MHR levels are associated with the severity of coronary artery disease. We think that adding these easily and quickly measurable parameters to routine laboratory results may support the clinician in evaluating the complexity of coronary artery disease and guiding treatment decisions in NSTEMI patients.

## 1. Introduction

Coronary artery disease (CAD) is one of the leading causes of death in the world. Cardiovascular diseases are responsible for 45% of deaths in women and 38% of deaths in men under the age of 75 in Europe [1]. Unstable angina pectoris (UA), non-ST segment elevation myocardial infarction (NSTEMI), ST segment elevation myocardial infarction (STEMI), and sudden cardiac death, which are within the spectrum of coronary artery disease ranging from stable ischemic heart disease to sudden cardiac death, are called acute coronary syndromes (ACS). UA is a type of ACS in which biological markers are not elevated and there is no pathological ST segment elevation. NSTEMI is characterized by an increase in biological markers but no pathological ST segment elevation. STEMI is recognized by ST segment elevation and an increase in biochemical and hematological markers [2].

Neutrophils, monocytes, and lymphocytes serve as an important part of the innate immune system and play an active role in processes associated with endogenous inflammation. Inflammatory cells at the site of atherosclerotic lesions have an important pathophysiological role in plaque rupture [3]. Circulating monocytes, which are the source of various cytokines and molecules, play a role in inflammatory and prothrombotic processes by interacting with platelets and endothelial cells [4]. These cells are also one of the most important components of the inflammatory process in atherosclerotic plaques. A high monocyte count during the acute phase of acute myocardial infarction (AMI) has been associated with plaque progression [5]. Additionally, monocytosis has been found to be an independent marker for coronary artery disease (CAD) and AMI. Neutrophil-to-lymphocyte ratio (NLR) and monocyte-to-lymphocyte ratio (MLR) were evaluated in studies conducted to show the severity of the disease and examine its prognostic value in CAD [5,6].

High-density lipoprotein cholesterol (HDL-C) protects endothelial cells by aiding vasorelaxation, interfering with low-density lipoprotein cholesterol oxidation and increasing endothelial nitric oxide synthase expression [7]. Recently, monocyte-to-HDL-C ratio (MHR) has emerged as a new marker in cardiovascular diseases, combining the predictive and prognostic value of easily available laboratory markers in a single fraction [8]. So, MHR combines oxidative stress due to the pro-inflammatory effect of monocytes and the antioxidant and anti-inflammatory effect of HDL-C [9]. Recent studies have demonstrated the relationship between MHR and ACS [10]. In the ACS patient population, MHR has been shown to be a predictor of stent thrombosis and in-hospital major cardiac events as well as mortality [10]. In a study, it was reported that there is a relationship between MHR and atherosclerotic vessel prevalence and atherosclerosis burden [11].

Currently, the most important biomarker used in the diagnosis of ACS is the serum concentration of cardiac troponin. Serum concentration of cardiac troponins may be useful not only in the diagnosis of acute cardiovascular events but also in the screening evaluation of the cardiovascular system [12]. Diagnosing ACS at an early stage and starting treatment will reduce morbidity and mortality, as well as providing easier control of complications that may arise. Therefore, risk scoring systems and biochemical markers are important factors in both early diagnosis and evaluation of short- and long-term prognosis. SYNTAX score (SS), an anatomical scoring system, is used to determine the complexity, morphology, prevalence, and severity of lesions based on coronary angiography based on their location in the coronary artery system [13]. After calculating the SS, the SS-II is created by adding some clinical features [14,15]. SS-II is designed to improve decision-making between CABG and PCI by allowing a long-term personalized risk assessment of patients with complex coronary artery disease.

In this study, we aimed to evaluate socio-demographic data, NLR, MLR, and MHR of patients with NSTEMI according to the values of the SS-II.

## 2. Materials and Methods

### 2.1. Study Design and Study Population

This study was approved by the decision of the Ethics Committee of the Health Sciences University dated 12 December 2023 and numbered 2023/454. The Declaration of Helsinki protocol was followed in the research protocol. Our study is a retrospective study. This study was conducted by selecting consecutive patients who were admitted to the outpatient clinic and emergency department at the private Aktif International Hospital Cardiology Department between 1 January 2020 and 31 December 2023.

The diagnosis of NSTEMI was made according to the 2023 ESC Guidelines for the management of ACS. The diagnosis was based on clinical symptoms, ECG findings, and elevated levels of cardiac biomarkers such as troponin in the absence of ST-elevation on the ECG [1].

**Figure 1:**
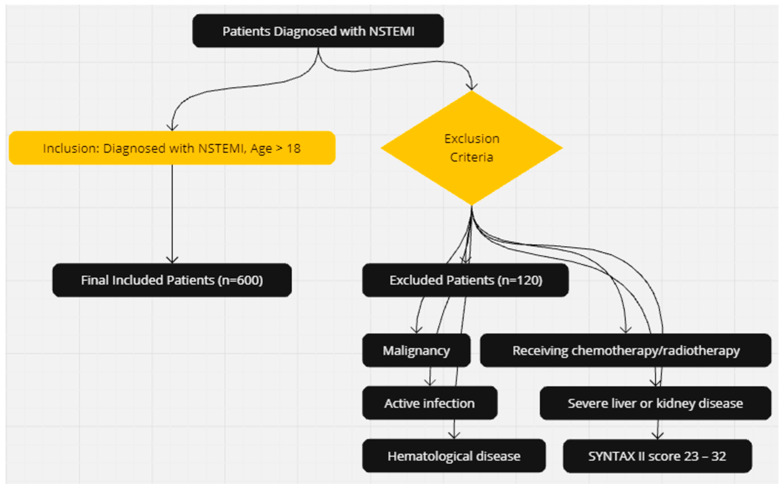
Study Design and Study Population. NSTEMI, Non-ST-elevation myocardial infarction.

The inclusion criteria for the study were patients diagnosed with NSTEMI over the age of 18. Exclusions from the study were patients with SS-II = 23–32, diagnosed with malignancy, severe liver or kidney disease, active infection, receiving chemotherapy/radiotherapy, and hematological disease. After exclusion criteria (*n* = 120), a total of 600 patients diagnosed with NSTEMI were included in the study (Figure 1).

Our study was designed as single-center, retrospective, and cross-sectional. Patients diagnosed with NSTEMI were included in the study. The baseline routine blood tests of these patients after their admission to the cardiology intensive care unit were scanned and recorded through a retrospective system. Socio-demographic data, complete blood count (CBC), hemogram, hematocrit, white blood cell, monocyte and lymphocyte count, TG, TC, LDL-C, HDL-C level, CRP, creatinine, creatinine clearance, NLR, MLR, MHR, and platelet count data were obtained in the CBC.

Patients included in the study were treated according to current NSTEMI guidelines. Based on clinical evaluations, a combination of medical management and revascularization therapies was applied. Out of the total, 70% (420 patients) underwent PCI, while 10% (60 patients) underwent CABG due to more complex coronary anatomy as indicated by their SS-II. The remaining 20% (120 patients) received conservative medical treatment either due to patient preference, comorbidities, or lack of indication for revascularization.

SS-II calculation is made after calculating the SS score, taking into account age, gender, creatinine clearance, left ventricular ejection fraction (LVEF), presence or absence of major coronary lesion, chronic obstructive pulmonary disease (COPD), and PAD [16]. This scoring gives the estimated 4-year mortality if the patient undergoes PCI or CABG [14].

In studies, the SS-II is generally classified with the following threshold values: Low Risk: SS-II ≤ 22, Medium Risk: SS-II = 23–32, and High Risk: SS-II > 32 [17,18]. In our study, we evaluated only low-risk S-II and high-risk SS-II patients. Patients in the intermediate compartment (23–32) of the Syntax II score generally constitute a heterogeneous risk group and may negatively affect clinical decision-making processes. We did not include the intermediate compartment in our study in order to obtain more homogeneous risk profiles and to make clearer and more consistent decisions. Not including the middle section made the analyses simpler and more understandable. Therefore, not including the intermediate risk group contributed to the more robust and reliable results obtained. As a result, the reason why we did not include the intermediate compartment (23–32) of SS-II in our study was to create more homogeneous and distinct risk groups in clinical applications, to increase the clarity of the analyses, and to ensure the reliability of the results obtained.

### 2.2. Statistical Analysis

The Statistical Package for Social Sciences (SPSS) v.27 program (IBM, Armonk, NY, USA) was used to analyze the data. Kolmogorov–Smirnov and Shapiro–Wilk tests were used to determine the normality of continuous variables. Descriptive statistics are expressed as mean ± standard deviation for continuous variables and as a percentage for categorical variables. Continuous variables with normal distribution were compared using Student’s *t* test, and continuous variables with non-normal distribution were compared using the Mann–Whitney *U* test. Categorical variables were compared using the chi-square or Fisher’s exact test, where applicable. Pearson correlation analysis was used to evaluate the linear correlation between NLR, MLR, MHR, and SS-II. Multivariable logistic regression analysis was performed in order to determine the predictive capacity of high-risk NSTEMI (SS-II > 32). For the multivariable logistic regression analysis, covariates were selected based on clinical relevance and prior evidence from the literature [5,9,11,19,20]. The predictive capacity of high-risk NSTEMI (SS-II > 32) was determined using multivariable logistic regression, adjusting for relevant covariates such as age, gender, creatinine clearance, LVEF, and the presence of comorbidities (e.g., COPD, PAD). The results are reported as odds ratios (OR) with corresponding 95% confidence intervals (CI). The predictive capacity of individual markers (NLR, MLR, MHR) for high-risk NSTEMI (SS-II > 32) was evaluated by calculating the area under the curve (AUC) of the ROC curve. Results for *p* < 0.05 were considered statistically significant.

## 3. Results

Five hundred patients were included in the study and these patients were divided between group 1 and group 2 according to the Syntax score II. Group 1 (SS-II ≤ 22) included 380 patients, and group 2 (SS-II > 32) included 220 patients. While the average age of group 1 was 67.0 ± 7.2, the average age of group 2 was 71.50 ± 6.3. No difference was observed between the groups in terms of age and gender (*p* > 0.05). Among the socio-demographic data, the prevalence of smoking, HL, DM, and PAH were significantly higher in group 2 than in group 1 (*p* < 0.05). There was no difference between the groups in terms of COPD and HT (*p* > 0.05). There was a statistically significant difference in HDL, creatine value, white blood cell, troponin I, hs-TnT, and monocyte values in group 2 compared with group 1 (*p* = 0.001, *p* = 0.018, *p* = 0.031, and *p* = 0.001, respectively). NLR, MLR, MHR, and SS II values were statistically significantly higher in group 2 compared with group 1 (*p* = 0.015, *p* = 0.002, *p* = 0.001, and *p* = 0.001, respectively) (Table 1).

The risk factors were found to be significantly associated with high-risk NSTEMI (SS-II > 32) in the multivariable logistic regression analysis, including PAD (OR: 3.028, *p* = 0.040), troponin I (OR: 3.575, *p* = 0.015), hs-TnT (OR: 4.221, *p* = 0.010), NLR (OR: 4.528, *p* = 0.024), MLR (OR: 5.248, *p* = 0.012), and MHR (OR: 7.122, *p* = 0.010) (Table 2).

**Figure 2:**
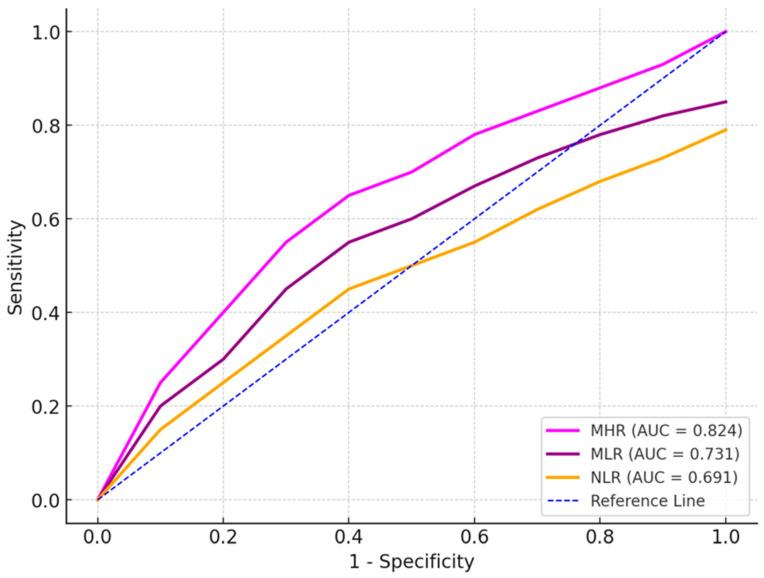
ROC analysis of parameters for cases with high-risk NSTEMI (SS-II > 32).

ROC analysis results in patients with high-risk NSTEMI (SS-II > 32) are shown in Table 3. ROC analysis revealed that NLR (AUC: 0.691, *p* = 0.016), MLR (AUC: 0.731, *p* = 0.004), and MHR (AUC: 0.824, *p* = 0.003) had higher predictive power than other parameters in patients with high-risk NSTEMI (SS-II > 32) (Table 3, Figure 2).

When we evaluated the linear correlation between inflammatory markers NLR, MLR, and MHR with SS-II in NSTEMI patients, there was a significant positive correlation between NLR and the SS-II (r = 0.650, *p* < 0.001), suggesting that higher NLR values are associated with greater coronary complexity. Similarly, the MLR demonstrated a moderate positive correlation with the SS-II (r = 0.707, *p* = 0.002), supporting its predictive value in assessing coronary artery disease severity. Lastly, the MHR also showed a positive correlation with the SS-II (r = 0.825, *p* = 0.001), further underscoring the role of inflammatory and lipid markers in predicting coronary complexity.

## 4. Discussion

There are a limited number of studies in the literature evaluating the relationship between biochemical markers and SS-II in NSTEMI patients. In this study, we planned to show the relationship between comorbidities and laboratory parameters and SS-II in patients with NSTEMI, and thus to reveal the presence and severity of coronary artery disease in patients with NSTEMI. The strength of our study is that it is a study that produces a more meaningful result by evaluating the important parameters of NSTEMI patients, which are evaluated individually in the literature, in a single study. We found that NLR, MLR, MHR, and PAD had a significant relationship with SS-II, which measures the severity and extent of coronary artery disease, in patients diagnosed with NSTEMI and receiving interventional treatment. The results of our study are compatible with a limited number of studies in the literature. Considering the studies on SS-II, which have not yet reached a sufficient level in current studies, we think that our study will provide additional information to the current literature.

Many previous studies have shown that inflammatory indicators (IL-6, IL-1, TNFα, and CRP) increase in atherosclerotic cardiovascular heart diseases [21,22,23,24,25]. If inflammatory responses are reduced, there is a slowdown in the atherosclerotic process and a subsequent reduction in cardiovascular events [21]. It is crucial to highlight that, as recently demonstrated, inflammation can contribute to destabilizing atherosclerotic plaques and cause future cardiovascular events even in patients with myocardial infarction with non-obstructive coronary arteries [26]. Recently, it has been thought that the number of white blood cells and leukocyte subtype cells found in the complete blood count play a role in predicting the inflammatory process in atherosclerotic cardiovascular heart diseases [21]. Clinical studies have found an independent association between deaths in AMI and increased WBC count [22]. It has been shown that neutrophils, the most abundant WBC subtype in the blood, play an important role in the inflammatory responses of cells [22]. While the number of neutrophils in atherosclerotic lesions is inversely proportional to the fibrous sheath thickness and number of smooth muscle cells, it has been shown to be directly proportional to the necrotic core area, plaque sensitivity, and the size of the atherosclerotic lesion. Increased neutrophil count in AMI has been associated with leukocyte platelet aggregation in the microcirculation leading to no reflow phenomenon, increased infarct area, unfavorable angiographic outcomes, and short-term prognosis in both NSTEMI and STEMI [23,24,25]. In our study, WBC was significantly higher in the group with SS-II > 32, while neutrophil count and CRP levels were not significant, although they were high.

After lymphocyte cells undergo apoptosis, the atherosclerotic plaque grows, and a lipid core develops, resulting in rupture of the atherosclerotic plaque and subsequent thrombus development [27]. Lymphopenia is an indicator of poor prognosis in cardiovascular diseases such as heart failure, ACS, and stable CAD [28]. In our study, although the lymphocyte count was low in the group with SS-II > 32, it was not significant.

Monocyte cells affect many stages from the beginning of the atherosclerotic event to the release of procytokines. Monocytes taken into the intima layer include oxidized LDL and other lipid particles, and they turn into foam cells, resulting in the formation of atherosclerotic plaques. The number of monocytes in the blood has been shown to be a warning marker of new plaques developing and of existing high-volume plaques [29]. In a prospective cohort study, 951 patients were recruited and patients underwent elective CAG procedure. As a result, after 2.5 years of follow-up, monocyte cells were found to be an independent predictive indicator of stroke, MI, and death from cardiovascular causes [30]. In another study, as a result of a 15-year follow-up of a population of 700 people without any cardiac disease, it was shown that increased monocyte count had a high predictive value in terms of cardiovascular events, independent of age, gender, and comorbid diseases [31]. In our study, the monocyte count was significantly higher in the NSTEMI group with SS-II > 32. SS-II values > 32 were not predictive of NSTEMI.

After it was shown that the number of circulating blood cells such as neutrophils, lymphocytes, monocytes, and HDL could have a predictive value in terms of CAD, ratios of inflammatory values such as NLR, MLR, and MHR were brought to the fore. This has been the subject of many studies, considering that the ratios obtained will be more meaningful than evaluating the cells alone. In our study, we especially focused on these three parameters.

It has been observed that NLR values are an independent predictor of mortality in cardiovascular diseases such as ST-segment elevation myocardial infarction (STEMI), and that they predict mortality in patients hospitalized with a diagnosis of ACS [32]. Additionally, high NLR values have been shown to predict the severity of atherosclerosis in CAD and to correlate with SS scoring [33]. Kaya et al. determined the relationship between NLR level and atherosclerosis prevalence. At NLR 2.5 and above, its sensitivity was 62% and its specificity was 69%, and NLR was significantly higher in the prevalent atherosclerosis group than in the mild atherosclerosis and control groups (4.1 ± 3.0, 2.4 ± 1.2, and 1.9 ± 1.2, respectively) [32]. In another study by Azab et al., it was reported that NLR > 4.7 was a significant predictor in the short and long term in patients with NSTEMI [33]. What we really need to know about NLR is which ranges we will consider normal and after which range it has prognostic value. In general, significant prognostic values above a ratio of 2.5 to 5.0 have been reported in studies. However, there are studies conducted at intervals from 2.5 to 11. In an article published by Lee et al., limit values were investigated, and ratios of 5.0 and above were found to be significant [34]. In our study, the NLR value was significantly higher in the group with SS-II > 32, and, according to the ROC analysis, NLR > 5.90 levels were predictive of SS-II > 32. Additionally, NLR was found to be significantly associated with NSTEMI in the logistic regression analysis.

A limited number of studies suggest that MLR can be used as an inflammatory marker in NSTEMI [35,36]. Fan et al. studied patients with stable angina pectoris. As a result of the study, it was stated that MLR could help detect the presence of sensitive plaque in unstable angina [37]. Murat et al. determined that there was a significant relationship between the restenosis seen in bare metal stents implanted in stable CAD patients and the MLR level [38]. Liyis et al. reported that NLR and MLR values were not significant in predicting NSTEMI in 115 ACS patients [36]. Contrary to this study, in our study, NLR and MLR values were significantly higher in the group with NSTEMI and SS-II > 32, and according to the ROC analysis, MLR > 0.58 levels were predictive of SS-II > 32. Additionally, MLR was found to be significantly associated with NSTEMI in the logistic regression analysis.

MHR, a newly developed inflammatory parameter, consists of the ratio of monocyte count and HDL-cholesterol level. A limited number of studies suggest that MHR can be used as an inflammatory marker in NSTEMI [39,40]. Kanbay et al. stated that MHR was an independent prognostic factor of major cardiovascular events in the follow-up of chronic kidney disease patients [20]. Kundi et al. showed that there was a significant and independent correlation between SS and MHR in patients with stable CAD [8]. Açıkgöz et al. stated that MHR was associated with long- and short-term mortality in patients presenting with STEMI and treated with primary PCI [19]. Kalyoncuoglu et al. reported that MHR, LVEF, smoking, and SS values were predictive in NSTEMI patients [39]. In our study, LVEF and smoking were not predictive in NSTEMI patients. However, PAD was predictive in NSTEMI patients. In addition, the MHR value was significantly higher in the group with SS-II > 32, and, according to the ROC analysis, MHR > 0.080 levels were predictive of SS-II > 32. Additionally, MHR was found to be significantly associated with NSTEMI in the logistic regression analysis. In our study, the strongest predictive parameter for NSTEMI and SS-II > 32 was MHR.

In addition to the well-established role of inflammation in the pathophysiology of myocardial infarction, recent advancements in non-invasive imaging techniques have provided new insights into the assessment of myocardial inflammation and infarct severity. Cardiac magnetic resonance imaging (CMR), in particular, has emerged as a valuable tool for evaluating myocardial inflammation and viability in patients with acute myocardial infarction. CMR can offer detailed assessments of both the infarcted and remote myocardium, including the extent of edema and scar formation, which are key predictors of long-term outcomes. Furthermore, CMR can identify myocardial areas at risk and provide a comprehensive view of the severity of the infarction, aiding in risk stratification and prognosis [41]. Incorporating CMR into the diagnostic and prognostic evaluation of patients with myocardial infarction may improve patient management by providing critical insights into the extent of myocardial injury and inflammation.

### Limitations of the Study

There are some limitations in our study. The fact that it is a single-center, small group of patients, and retrospective design study limits the comprehensiveness of the data due to the nature of the study. We did not include the intermediate compartment in our study in order to obtain more homogeneous risk profiles and to make clearer and more consistent decisions. Not including the middle section made the analyses simpler and more understandable. Moreover, the strength of our study is that it is a study that produces a more meaningful result by evaluating the important parameters of NSTEMI patients, which are evaluated individually in the literature, in a single study.

## 5. Conclusions

In conclusion, we found that NLR, MLR, MHR, and PAD had a significant relationship with SS-II, which measures the severity and extent of coronary artery disease, in patients diagnosed with NSTEMI and receiving interventional treatment. The results of our study are compatible with a limited number of studies in the literature. Our study provides additional information to the current literature.

Although NLR, MLR, and MHR are simple, inexpensive, and easy-to-measure tests, their association with the severity of coronary artery disease and their utility in predicting short-term mortality and morbidity in NSTEMI patients require further validation in larger multicenter studies.

## Figures and Tables

**Table 1:** Comparison of patients’ socio-demographic, clinical, and laboratory parameters.

Parameter	Group 1, SS-II ≤ 22 (N = 380) Mean ± SD; *n* (%)	Group 2, SS-II > 32 (N = 220) Mean ± SD; *n* (%)	*p*
Age (year)	67.0 ± 7.2	71.50 ± 6.3	0.906
Gender Male Female	108 (38.8%) 172 (62.2%)	91 (41.6%) 129 (58.4%)	0.295
Smoking	56 (20.0%)	128 (58.3%)	0.001 *
Hypertension (HT)	180 (64.4%)	143 (65.0%)	0.194
Hyperlipidemia (HL)	31 (11.1%)	66 (30.0%)	0.022 *
Diabetes mellitus (DM)	109 (38.8%)	111 (50.3%)	0.030 *
COPD	37 (13.3%)	38 (17.3%)	0.381
Peripheral artery disease (PAD)	53 (18.8%)	73 (33.3%)	0.001 *
Hemoglobin (g/dL)	11.13 ± 1.7	10.95 ± 2.1	0.715
HDL (mg/dL)	43.1 ± 10.1	25.2 ± 9.1	0.001 *
LDL (mg/dL)	137.2 ± 31.2	154.1 ± 34.4	0.698
Total cholesterol (mg/dL)	182.5 ± 42.9	198.6 ± 45.2	0.492
Triglycerides (mg/dL)	157.1 ± 49.4	165.6 ± 55.0	0.922
CRP (mg/dL)	44.53 ± 20.7	64.92 ± 34.8	0.065
Creatinine (mg/dL)	1.00 ± 0.8	1.20 ± 1.0	0.018 *
Creatine Clearance (mL/min)	65.6 ± 15.3	78.6 ± 18.2	0.065
White Blood Cell (WBC) (10^3^/μL)	11.16 ± 3.7	13.48 ± 1.0	0.031
Neutrophils (10^9^/L)	7.87 ± 4.8	12.85 ± 4.8	0.051
Lymphocytes (10^9^/L)	3.08 ± 6.1	2.54 ± 4.8	0.667
Platelets (10^9^/L)	244.47 ± 69.7	293.64 ± 82.6	0.076
Monocytes (10^9^/L)	0.73 ± 0.31	2.06 ± 3.9	0.001 *
Troponin I (ng/mL)	0.2 ± 0.15	1.8 ± 1.2	0.001 *
hs-TnT (ng/L)	14.0 ± 8.0	56.0 ± 34.0	0.001 *
Neutrophil-to-lymphocyte ratio (NLR)	2.85 ± 2.6	6.92 ± 3.8	0.015 *
Monocyte-to-lymphocyte ratio (MLR)	0.520 ± 0.27	0.600 ± 0.54	0.002 *
Monocyte-to-HDL ratio (MHR)	0.029 ± 0.01	0.093 ± 0.17	0.001 *
LVEF (%)	55.9 ± 2.5	52.2 ± 2.2	0.058
Syntax score (SS)	16.0 ± 7.2	32.2 ± 4.1	0.001 *
Syntax II score (SS-II)	19.7 ± 2.2	35.3 ± 5.4	0.001 *

*: Student’s *t* test. COPD: Chronic obstructive pulmonary disease. HDL: High-density lipoprotein. LDL: Low-density lipoprotein. CRP: C-reactive protein. hs-TnT: High-sensitivity troponin T. LVEF: left ventricular ejection fraction.

**Table 2:** The effect of parameters in predicting high-risk NSTEMI (SS-II > 32).

Variable	Odds Ratio	95% CI	*p*
Hyperlipidemia (HL)	2.125	0.45–11.14	0.083
Diabetes mellitus (DM)	2.143	0.37–5.52	0.096
Peripheral artery disease (PAD)	3.028	2.02–4.07	0.040
HDL (mg/dL)	2.028	1.57–3.85	0.084
Creatinine (mg/dL)	0.900	0.98–82.49	0.373
WBC (10^3^/μL)	0.606	0.29–1.24	0.453
Monocytes (10^9^/L)	2.125	0.45–11.14	0.093
Troponin I	3.575	1.51–8.02	0.015
hs-TnT	4.221	1.80–9.65	0.010
Neutrophil-to-lymphocyte ratio (NLR)	4.528	2.67–8.55	0.024
Monocyte-to-lymphocyte ratio (MLR)	5.248	1.76–14.64	0.012
Monocyte-to-HDL ratio (MHR)	7.122	1.29–15.22	0.010

**Table 3:** ROC analysis results in patients with high-risk NSTEMI (SS-II > 32).

	Cut-Off	Sensitivity	Specificity	AUC (95% CI)	*p* Value
Neutrophil-to-lymphocyte ratio (NLR)	>5.90	59.0%	56.7%	0.691 (0.385–0.597)	0.016
Monocyte-to-lymphocyte ratio (MLR)	>0.580	69.0%	66.7%	0.731 (0.545–0.717)	0.004
Monocyte-to-HDL ratio (MHR)	>0.080	73.1%	70.0%	0.824 (0.624–0.893)	0.003

## Data Availability

The original contributions presented in the study are included in the article, further inquiries can be directed to the corresponding authors.
